# Developing a shortened version of the dementia knowledge assessment scale (DKAS-TC) with a sample in Taiwan: an item response theory approach

**DOI:** 10.1186/s12877-022-03596-1

**Published:** 2022-11-22

**Authors:** Su-Pin Hung, Yi-Han Liao, Claire Eccleston, Li-Jung Elizabeth Ku

**Affiliations:** 1 Center of Teacher Education, National Cheng Kung University, Tainan, Taiwan; 2 Institute of Education, National Cheng Kung University, Tainan, Taiwan; 3 Department of Public Health, College of Medicine, National Cheng Kung University, No.1, University Road, Tainan City, 701 Taiwan; 4 Wicking Dementia Research and Education Centre, University of Tasmania, Hobart, Australia

**Keywords:** Dementia, Knowledge, Item response theory, Shortened scales, Psychometrics

## Abstract

**Background:**

The 25-item Dementia Knowledge Assessment Scale (DKAS2) is a widely used tool for measuring knowledge of dementia. To increase the applicability of the Chinese-language version of the tool (DKAS-TC) for the general public, this study aimed to develop a shortened version using the item response theory (IRT) approach.

**Methods:**

A total of 401 participants voluntarily completed a Chinese-language version of the DKAS2 questionnaire (DKAS-TC) at the start of dementia awareness training courses in 2020 and 2021. The four Rasch family models were used to analyze the dimensionality of the shortened scale (the DKAS-s) and to confirm its accuracy in measuring dementia knowledge.

**Results:**

The results justified supported the use of a dichotomous response scale for responding to the DKAS-s and demonstrated good fit of the data to a Rasch model with the four dimensions of “Causes and Characteristics”, “Communication and Engagement”, “Care Needs”, and “Risks and Health Promotion”. Moreover, we shortened the DKAS-TC by selecting items that had both above-average discriminative ability and above-average information. The DKAS-s retained 64.13% of the information contained in the DKAS-TC, resulting in a 16-item scale which retained four items in each of the original four dimensions. The DKAS-s also correlated highly (≥0.95) with the DKAS-TC and exhibited a sizeable range of difficulty of dementia knowledge.

**Conclusions:**

The DKAS-s is expected to be more efficient in field settings while retaining an acceptable level of psychometric properties when used as a survey instrument to measure the general public’s knowledge of dementia.

**Supplementary Information:**

The online version contains supplementary material available at 10.1186/s12877-022-03596-1.

## Background

The prevalence of dementia has increased dramatically with the aging of the global population. In 2021, the percentage of seniors 65 or older in Taiwan is 16.6% [1], and the number of people living with dementia had exceeded 300,000 by the end of 2020, accounting for 1.29% of the entire population of Taiwan [2]. The Taiwanese government launched the “Taiwan Dementia Policy: A Framework for Prevention and Care” in 2014, with the goal of achieving more than 5% of Taiwanese citizens with an awareness of dementia [3]. However, the government did not provide a specific definition of “awareness of dementia”, nor was a proper tool made available to measure the general public’s knowledge of dementia. Therefore, there remains a need to develop a suitable measurement instrument for evaluating the knowledge of dementia in the Chinese language.

According to systematically conducted reviews published in the past 5 years, there is no consistently used measurement tool for assessing public knowledge of dementia [4, 5]. Among the examples of such instruments identified in a scoping review, the most frequently used was the Alzheimer’s Disease Knowledge Scale (ADKS) [6], followed by the Dementia Knowledge Assessment Tool Version 2 (DKAT2) [7]. Although scales including ADKS [6], Knowledge of Memory Loss and Care Test [8], and the Dementia Quiz [9] have been developed to assess knowledge of dementia, they have been used with small samples or a specific population at a particular developmental stage, resulting in a lack of generalizability [10]. Furthermore, the three aforementioned scales have mostly focused on the biomedical domain or specific types of dementia, which may lead to a ceiling effect for educated members of the general public. Such shortcomings highlight the need for the use of more robust, contemporary knowledge assessment tools applied to the evaluation of the effectiveness of psychosocial interventions related to dementia [11].

As one contemporary tool developed by Annear et al. in 2016, the Dementia Knowledge Assessment Scale (DKAS) was an improvement of the DKAT2 in order to address the limitations of existing measurement instruments [10, 12]. The DKAS includes four components, defined as “Causes and Characteristics”, “Communication and Engagement”, “Care Needs”, and “Risks and Health Promotion”. It has been shown to be a reliable, valid measure of dementia knowledge and to perform better than the ADKS when administered to a large and diverse international cohort [12]. It was shortened to a 25-item version (DKAS2) after being evaluated with a confirmatory factor analysis (CFA) [13]. The original response options for each item included “true”, “possibly true”, “false”, “possibly false”, and “don’t know”, but the scoring system required recording responses to “fully correct” (2), “partly correct” (1), and “incorrect” (0), with a total score ranging from 0 to 50.

The DKAS2 has been translated into Chinese by different research teams. There are currently three translated versions, including one using traditional Chinese characters [14] and another one with simplified Chinese characters [15]. The third one, also using traditional Chinese characters (the DKAS-TC), was developed by Sung et al. and is the version of the questionnaire we used in this study [16]. In fact, there are considerable differences in the three Chinese-language versions of the DKAS2 in terms of the wordings of the test items and the scoring methods.

The first of these translated assessment tools is a traditional Chinese version of the DKAS2 which has been translated by Taiwanese scholars for use in Taiwan (the T-DKAS) [14]. The participants were 150 adults who were administered the T-DKAS in order to determine its level of reliability (a Cronbach’s alpha of 0.78). The scoring method was different from that of the original version, with the response to each item recorded as “incorrect” or “correct” (score = 0 or 1, respectively) so that the total score of the T-DKAS was 25 [14].

Another study was conducted in China to verify the psychometric properties of a simplified Chinese version of the DKAS2 questionnaire (the DKAS-C) [15]. The respondents included 290 healthcare providers recruited from care homes and hospitals. The Cronbach’s alpha of the DKAS-C was 0.77, indicating that the DKAS-C has good reliability. It was also determined that the DKAS-C had an acceptable level of concurrent validity by finding a moderate correlation between the DKAS-C and the Chinese-language version of the ADKS [15].

Our study used the third traditional Chinese-language translation of the DKAS2, also known as the DKAS-TC [16]. The results of the CFA confirmed the validity of the use of the four-factor, 25-item model for the DKAS-TC. The DKAS-TC also achieved a good overall Cronbach’s alpha of .93 and McDonald’s omega of 0.94. In addition to having good reliability, another important factor behind our choice to use this scale was that the meaning of the translated words is closer to the English used in the original questionnaire and also for cultural relevance. For instance, the DKAS-C and the DKAS-TC used different vocabularies for translating the term “depression” reflecting different cultural traditions between Taiwan and China. Although the DKAS-TC has shown good psychometric properties, their participants were limited to healthcare providers [16] . Therefore, this study aimed to determine whether their questionnaire is applicable to our target population: the general public.

Recently, item response theory (IRT) based methods have been used widely across disciplines in the social, behavioral, and health sciences [17–22]. IRT offers several advantages over classical test theory (CTT) for questionnaire design [23, 24]. One of the advantages of IRT over CTT is the unbiased estimates of item properties even from two extreme samples. Moreover, the basis of accurate estimation of measurement is different between IRT and the CTT. A constant standard error is assumed in CTT regardless of a respondent’s test total score. Instead, in IRT models, the standard error of measurement differs across scores and generalizes across populations. It provides a precise estimation for the latent trait of the measurement. Once item parameters are estimated, the test information function indicates “measurement precision” of the scale in measuring the construct of interest [23, 25, 26]. Item and test information have many uses in IRT: item information is used to select items for inclusion, and test information can be used to compare two competing measures of a construct [24]. Previous studies have demonstrated how the IRT provides powerful tools for item selection and scale shortening [27–29]; for example, one study applied IRT analysis to obtain a 10-item scale from the original 18-item Need for Cognition Scale (NFC-18) while maintaining the measurement’s reliability and validity [27].

These features, among others, along with the drawbacks inherent in CTT, have led us to apply the IRT approach to shortening the Chinese-language version (DKAS-TC) of one of the frequently used measures of dementia awareness —the 25-item DKAS2. This study aimed to develop a short-form of DKAS-TC, hereafter referred to as the DKAS-s, to increase the applicability of the test items and shorten the time required to answer the questionnaire.

## Methods

### Participants and material

All participants of this study completed the DKAS-TC questionnaire at the start of dementia awareness-raising training courses taught by the corresponding author. The sample consisted of three kinds of participants attending the course at different times: bank employees (103 people), undergraduate students (201 people), and pharmacists (137 people). The bank employees were recruited from two financial institutions working in local branches throughout Taiwan. The undergraduate students were enrolled in an introduction to public health course at a national university in southern Taiwan. The pharmacists were recruited through public health-education training programs conducted in Tainan City, in southern Taiwan. The training course for bank employees were held as part of a funded project into the financial security of persons with dementia, but the other two respondent groups came from purposive sampling of participants in a dementia awareness course taught by the same instructor. Data collections complied with all relevant ethical regulations and was approved by the Institutional Review Board of the National Cheng Kung University Hospital (No. A-ER-108-101).

The participants agreed to complete the DKAS-TC anonymously. Between September 2020 and May 2021, 441 participants were invited to participate in the questionnaires and all agreed to undertake the pre-class survey. Incomplete questionnaire responses which amounted to 4.7% of the data were excluded from the analysis. Therefore, a total of 410 valid questionnaire responses were collected in class, of which the undergraduate students accounted for the largest part (43%), followed by the pharmacists (32%) and the bankers (25%). There were slightly more female participants (54%) than male participants. As for the format of the survey, since the undergraduate students usually took their class quizzes online, this survey was also completed online, while bank employees and pharmacists filled out paper questionnaires.

In addition to the completing the DKAS-TC, our participants also provided demographic information, including their gender, age, and education level. A summary of participant demographics is presented in Additional file 1: Appendix 1. As shown in the Additional file 1: Appendix 1 Table, results from Chi-square test showed that the age distribution across the three professional groups were significantly different, with undergraduates as the youngest group, but results for gender distribution was similar across groups.

### Data analysis

Because the original DKAS2 adopted partial credit scoring of responses (0 = “completely don’t know/wrong answer”; 1 = “partially correct”; and 2 = “completely correct”), the polytomous partial credit model (PCM) [30] was used to represent respondents’ knowledge and partial knowledge of dementia. The partial credit scoring intended to allow respondents who present partial knowledge of dementia on each endorsed item. For simplicity this is referred to herein as the PCM. Additionally, a dichotomous scoring Rasch model [31], referred to herein as the Rasch model, was tested to reflect the correct compared with incorrect knowledge of dementia of the respondents. This considers items in the original DKAS2 to require dementia-related factual decisions and justifies the assumption that there is no ‘partially’ correct answer. With the Rasch model, both the “completely wrong” response and the “partially correct” response were recoded as “wrong” responses. The PCM belongs to the Rasch family of models, and the four models tested in this study were: a unidimensional PCM, a four-dimensional PCM, a unidimensional Rasch, and a four-dimensional Rasch model. Maximum marginal likelihood methods of estimation were employed for item parameter estimation. Model-data fit was assessed using the full information in the test by means of the − 2 loglikelihood (−2*LL*) statistic, Akaike information criterion (AIC) [32], Bayesian information criterion (BIC) [33], as well as the root mean square error of approximation (RMSEA) [34, 35]. Specifically, the difference in -*2LL*s between the two competing models is distributed as a chi-square and, thus, allows for statistical comparisons between models. For the global measure of model fit such as AIC and BIC statistics, the lower the value, the better the model fits the data. Note the RMSEA is calculated using the M2 statistics which uses second-order marginal probabilities rather than using the full information in the test. The RMSEA values below 0.05 was applied to identify the best data-model fit [36].

The expectation of the marginal posterior distribution, or the expected a posteriori (EAP) estimate [37], was used to generate predictions of the latent trait scores of the respondents. To identify undesired and problematic response patterns, the weighted (infit) mean-square fit statistic (MNSQ) and the unweighted (outfit) MNSQ were adopted to assess item quality. The ideal value of both the infit and outfit MNSQ is 1.0, but the acceptable values of these statistics generally fall in the range of 0.5–1.5 [38]. All analyses were conducted using the ConQuest 3.0.1 computer program [39] and R Statistical Software (v4.2.0; R Core Team 2022) with the mirt package [40].

To verify the effectiveness of the DKAS-TC, both item separation reliability (ISR) and person separation reliability (PSR) were calculated [41]. The PSR is interpreted in the same way as the Cronbach’s alpha [17], and > 0.70 indicates adequate reliability. Additionally, both item and test information functions graphically reflect the reliability of the individual items and how the test as a whole estimates the construct being assessed over the entire scale range.

Along with the fit indexes based on IRT, we also considered results based on item-to-total correlations (≧0.3 and < 0.8 were considered acceptable) and measures of internal consistency (obtained with Cronbach’s alpha) [42] to evaluate item quality and assess the appropriateness of scoring items together on a single scale (α ≥ 0.70 was considered acceptable).

## Results

### Stage 1: elucidate the dimensionality and scaling of the DKAS-TC

To investigate the dimensionality and scaling issues of the DKAS-TC, four models were employed and compared—the unidimensional Rasch, the four-dimensional Rasch, the unidimensional PCM, and the four-dimensional PCM model. As shown in Table 1, the four-dimensional Rasch model demonstrated better fit to the data (−*2LL* = 5446.330; RMSEA = 0.058; BIC = 11,103.23; AIC = 10,962.66), which indicates that the four dimensions of the DKAS fit the observed data well, and thus item parameters discussed subsequently were obtained with the four-dimensional Rasch model. This finding provides theoretical support, on the one hand, that the DKAS measures the four dimensions of dementia knowledge it was designed to evaluate and, on the other, that responses to the DKAS can be given on a dichotomous response scale.

**Table 1:** Comparisons of unidimensional and 4-dimensional Rasch models and PCMs

Models	Deviance	Parameters	-2LL	AIC	BIC	RMSEA
Unidimensional Rasch model	11,023.44	26	− 5511.55	11,075.44	11,179.52	0.070
4-dimensional Rasch model	10,889.01	25	−5446.33	10,939.02	11,103.23	0.058
Unidimensional PCM	18,452.79	51	− 9226.40	18,554.79	18,759.62	0.071
4-dimensional PCM	18,324.44	60	− 9160.93	18,444.44	1862.84	0.072

*Abbreviations*: *PCM* partial credit model, −2*LL* 2 loglikelihood, *AIC* Akaike information criterion, *BIC* Bayesian information criterion, *RMSEA* root mean square error of approximation

Table 2 lists the descriptive statistics of the four-dimensional Rasch model of the 25-item DKAS-TC. The item-total correlation ranged from .20 to .58, which indicates considerable variation in the items’ discriminative ability. The Cronbach’s alpha reliability coefficient for the DKAS was .70. We also calculated Cronbach’s alpha if an item is deleted, which estimates what Cronbach’s alpha would have been if an individual item had been removed from the scale. Table 2 shows that none of these values were greater than .70, which suggests that all items can be included in the scale. The percentage of responses for each item in Table 2 suggests that most participants responded to the DKAS-TC items with either “correct” (2) or “incorrect” (0), whereas participants selected “partially correct” (1) the least often among all options.

**Table 2:** Basic descriptive statistics and parameter estimations based on the 4-dimensional Rasch model of the DKAS-TC

			Cronbach’s alpha = .70	Percentage of responses (%)^a^			Difficulty				Information Total Test = 123.809		
Dimension	Item No.	Item-total correlation	If item deleted alpha	Score			estimate	error	out FIT	In FIT	Value	%	PSR
				0	1	2							
Cause and characteristics	1	0.33	0.69	51.47	14.95	33.58	0.86	0.12	0.98	1.02	4.97	4.01%	0.74
	2	0.29	0.69	5.87	28.12	66.01	−0.83	0.12	1.08	1.02	4.97	4.01%	
	3	0.44	0.68	22.60	29.48	47.91	0.11	0.11	0.9	0.92	4.98	4.02%	
	4	0.36	0.69	21.08	19.12	59.80	−0.49	0.11	1.06	1.04	4.97	4.02%	
	5	0.23	0.69	4.39	8.05	87.56	−2.38	0.16	1.45	1.09	4.88	3.94%	
	6	0.30	0.69	69.46	17.24	13.30	2.29	0.16	0.78	0.97	4.89	3.95%	
	7	0.38	0.69	54.03	17.85	28.12	1.18	0.12	1.01	0.98	4.96	4.01%	
Risks and health promotion	8	0.48	0.69	30.79	28.82	40.39	0.57	0.12	0.85	0.93	4.97	4.02%	0.81
	9	0.41	0.69	21.08	19.12	59.80	−0.54	0.12	1.05	1.04	4.97	4.02%	
	10	0.47	0.68	38.24	29.17	32.60	1.03	0.12	0.97	1.01	4.96	4.01%	
	11	0.43	0.68	5.88	23.53	70.59	−1.22	0.13	0.85	0.93	4.96	4.01%	
	12	0.48	0.68	11.22	16.59	72.20	−1.33	0.13	0.83	0.86	4.96	4.00	
	13	0.20	0.70	82.89	7.82	9.29	3.03	0.19	1.07	1.12	4.78	3.86	
Communication and behavior	14	0.31	0.69	25.86	29.06	45.07	0.27	0.12	1.24	1.15	4.98	4.02%	0.77
	15	0.26	0.69	54.05	18.18	27.76	1.27	0.13	1.08	1.08	4.96	4.00%	
	16	0.43	0.68	25.43	25.18	49.39	0.04	0.11	0.93	0.97	4.98	4.02%	
	17	0.48	0.68	28.61	38.14	33.25	0.94	0.12	0.91	0.97	4.97	4.01%	
	18	0.40	0.69	30.47	37.59	31.94	1.01	0.12	0.96	0.95	4.97	4.01%	
	19	0.34	0.69	48.16	23.59	28.26	1.24	0.13	0.93	1.01	4.96	4.00%	
Care considerations	20	0.46	0.68	9.11	18.97	71.92	−1.48	0.14	1.13	1.09	4.95	4.00%	0.78
	21	0.26	0.69	51.72	23.28	25.00	1.72	0.14	1.12	1.09	4.94	3.99%	
	22	0.55	0.68	10.02	30.81	59.17	−0.58	0.13	1.02	0.88	4.97	4.02%	
	23	0.36	0.69	20.54	29.58	49.88	0.02	0.13	1.01	0.98	4.98	4.02%	
	24	0.58	0.67	22.68	29.76	47.56	0.17	0.13	0.67	0.81	4.98	4.02%	
	25	0.39	0.69	13.20	28.12	58.68	−0.54	0.13	1.11	1.07	4.97	4.02%	

*Abbreviations*: *PSR* person separation reliability

^a^Percentage of respondents who responded on a three-point scale for each item

As for the assessment of item difficulty, estimations ranged from − 0.249 to 2.775, which suggests a sizeable range of difficulty of the underlying construct, the DKAS-TC. The model fit indices in Table 2 all fell between 0.5 and 1.5, indicating that all the DKAS-TC items fit the four-dimensional Rasch model well. Furthermore, the ISR of the scale was 0.989, which indicates the items were well distinguished by the test takers. On the other hand, the acceptable PSR for the four subscales (between 0.74 and 0.81) indicated that their items have acceptable internal consistency and so can be used to distinguish participants. These results justify the use of the four dimensions of the DKAS-TC.

### Stage 2: item selection for the DKAS-s

Both CTT-based and IRT-based item analyses for each item in the DKAS-TC were performed, and all the information available was carefully considered to decide on the best items to include in the final version of our shortened scale as well as their ideal number. First, we shortened the scale by selecting the items that had above-average discriminative ability (i.e. an item–total correlation value higher than 0.3). Thus, items 2, 5, 13, 15, and 21 were targets for removal because of having low item-total correlation values.

Second, we shortened the DKAS-TC by selecting the items that offered above-average information because an item with more information can more precisely differentiate the overall level of the DKAS-TC based on respondents’ ratings of individual items. The item information function, IIF, is an indication of item quality: of how much information an item provides about the IRT score. The test total information function, TIF, is a measure of the information provided by the item responses on a test. If the information value is high, it can be concluded that the extent to which an examinee’s performance represents his true ability at that point can be estimated with accuracy [43] [43]. For a 25-item scale, if each item is assumed to equally differentiate the level of the scale as a whole, each item should provide 4% of the total test information (i.e. 100% divided by 25 items) [44]. Therefore, we shortened the DKAS-TC by selecting the items that had better than average discriminative ability (i.e. providing more than 4% of the total test information). As shown in Table 2, items 5, 6, 13, and 21 provided less than 4% of the total test information and so were targets for removal.

Of the items examined under the first dimension of the DKAS-TC, we reserved item 6 instead of item 4 because the content of the latter requires medical knowledge to be clearly understood compared with the other items in the same dimension and so it may be misunderstood by the general public. Regarding the items in the second dimension, item 9 was deleted because its low degree of difficulty resulted in a negatively skewed score distribution, making it impossible to rule out the presence of a ceiling effect. As for the items in the third dimension, we kept the four items with the best item quality as measured by item-total correlation. Finally, in the case of the fourth dimension, item 25 was deleted for the same reason that item 4 was removed. As a result, in our DKAS-s, we retained four items in each dimension, all of which had acceptable infit and outfit MNSQs. The 16 items also reflected a sizeable range of difficulty of the underlying construct, as well as acceptable item information and PSR, as shown in Table 3.

**Table 3:** Results of parameters and information of the DKAS-s

						Information Total Test =79.397		
Dimension	Item No.	estimate	error	out FIT	In FIT	Value	%	PSR
Cause and characteristics	1	0.94	0.12	0.99	1.05	4.97	6.26%	0.66
	3	0.12	0.12	1.07	0.95	4.98	6.27%	
	6	2.44	0.16	0.74	0.92	4.89	6.16%	
	7	1.25	0.13	1.04	1.08	4.96	6.25%	
Risks and health promotion	8	0.65	0.13	0.89	1.01	4.97	6.26%	0.73
	10	1.17	0.13	0.92	1.07	4.96	6.25%	
	11	−1.37	0.14	1.05	0.99	4.96	6.25%	
	12	−1.47	0.14	1.03	0.96	4.96	6.24%	
Communication and behavior	16	0.05	0.12	1.07	1.04	4.98	6.27%	0.73
	17	1.00	0.12	0.82	0.9	4.97	6.26%	
	18	1.07	0.13	0.87	0.94	4.97	6.25%	
	19	1.33	0.13	1.07	1.08	4.96	6.25%	
Care considerations	20	−1.61	0.14	1.41	1.23	4.95	6.23%	0.80
	22	−0.63	0.13	1	1.05	4.97	6.26%	
	23	0.05	0.13	1.05	1.09	4.98	6.27%	
	24	0.19	0.13	0.77	0.95	4.98	6.27%	

*Abbreviations*: *PSR* person separation reliability

**Fig. 1:**
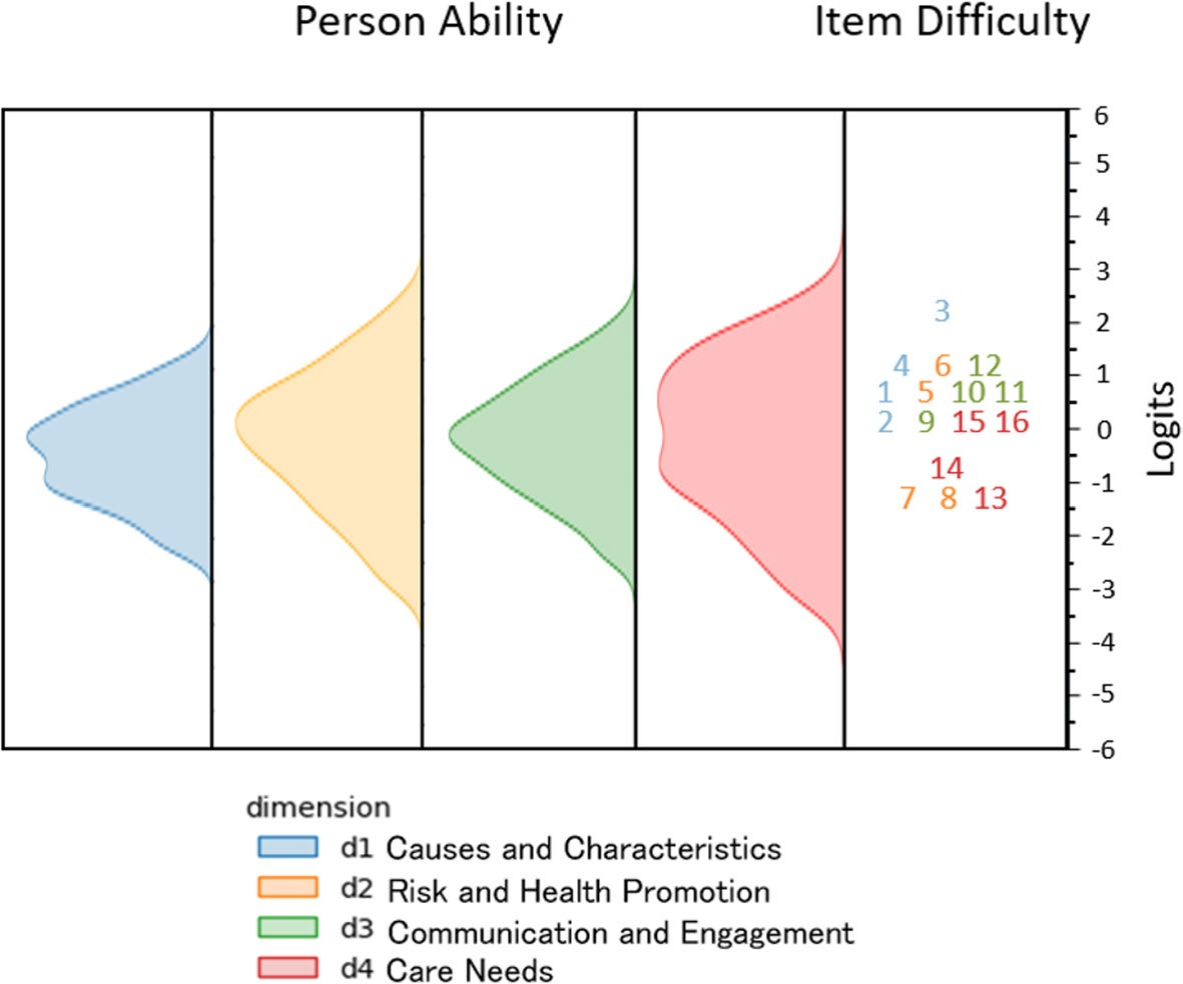
Item and person map for DKAS-s

Figure 1 shows the spread of item difficulty and the distribution of latent trait estimates. A participant whose DKAS-s latent trait estimate is the same as the level of difficulty of a given item has a 50% chance of endorsing this item. The “Xs” on the left-hand side of the figure represent the distribution of the participants’ ability on each dimension of the DKAS-s. The right-hand side shows the distribution of the item calibrations, with difficulty levels of the DKAS-s items ranging from − 1.61 to 2.44 logits. Ideally, the distribution of item difficulty levels would cover the entire span of the distribution of participants’ ability levels, thus providing an accurate measurement of participants’ proficiency over the whole scale. Both the results presented in Fig. 1 and the estimates of the item difficulty values shown in the Table 3 confirm that the DKAS-s items can locate most participants precisely. This is not the case for the participants who find themselves at the extreme ends of the scale, which indicates that additional items with greater difficulty and ease are needed for specific populations.

**Fig. 2:**
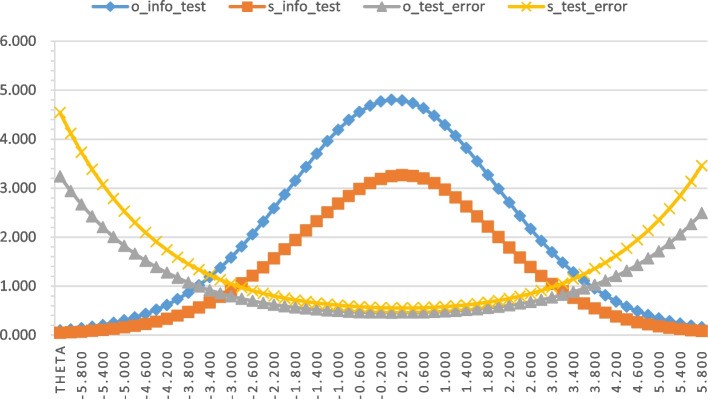
Test information and test error of original DKAS and DKAS-s. *Note:* Latent trait (Theta) is shown on the horizontal axis (higher values mean higher dementia knowledge); the amount of information and the estimate error yielded by the test at each trait level is shown on the vertical axis. o_info_test: test information of original DKAS, s_info_test: test information of short form DKAS-s, o_test_error: estimate error for latent trait level of original DKAS, s_test_error: estimate error for latent trait level of short form DKAS-s

In sum, our shorter version of the DKAS-TC retained 64.13% of the total test information of the original scale, which we determined by dividing the two values of Total Test Information. By aggregating the item information curves (IICs) of all the items in a scale, the test information function (TIF) curve can be generated [24]. As depicted in Fig. 2, the test information and test error of both the DKAS-TC and the DKAS-s provide sufficient information and spread the information over a wider range of ability levels.

### Preliminary validity evidence of the shortened scale

The correlation between the 25-item DKAS-TC and the shortened DKAS-s using their estimates of item difficulty is 1.00 (See Table 4), suggesting that the ordering of item difficulty of the two versions is identical. The bivariate Pearson correlation coefficients between the DKAS-TC and the DKAS-s using the estimates of EAP measures (i.e. the latent trait scores of the respondents on the four dimensions) are also listed in Table 4. For the complete 16-tem DKAS-s questionnaire and corresponding English translations, see Additional file 2: Appendix 2. All the correlations were greater than 0.95 and significant (*p* < 0.01). Given that the full-length DKAS-TC has proven to be predictive of important knowledge of dementia, it is assumed that the shortened DKAS-s is also significantly related to such knowledge. Results from additional MANOVA analyses showed that the highest DKAS-s item estimates were found among the pharmacists in all four domains when compared to both the college students and bank employees. This finding suggests that individuals from the health-allied sciences have higher ability in their knowledge about dementia.

**Table 4:** Pearson correlation coefficients between estimates for DKAS-TC and DKAS-s

		Dimension	DKAS-s^b^
DKAS-TC ^a^	Estimates of latent traits	Cause and characteristics	0.95
		Risks and health promotion	0.96
		Communication and behavior	0.95
		Care considerations	0.96
	Estimates of item difficulty		1

^a^A Traditional Chinese translation of the 25-item DKAS2

^b^A shortened version of the DKAS-TC

## Discussion

The primary goal of the current study was to develop an abbreviated Chinese-language version of the 25-item, four-dimensional DKAS-TC [16] for assessing the knowledge of dementia among the general public. The results of the series of IRT analyses we performed revealed that the items in the DKAS-s were able to differentiate between high and low levels of performance on the four dimensions of dementia knowledge. However, the item difficulty parameters indicated that, overall, the DKAS-s items were more useful for identifying populations with average DKAS-s scores than those with extremely high or low scores. We suggest that if the DKAS-s is administered to medical or nursing professionals, more difficult items should be added. Additionally, an interesting finding is that our study participants in Taiwan seemed to prefer the definitive responses of either “true” or “false” to the more vague or unsure answer of “possibly true/ false”. This observation is consistent both with our recommendation to use a dichotomous scale for responding to the DKAS items and the conclusion that the four-dimensional Rasch model has the best model fit. Furthermore, we argue that a simple dichotomous response format is more accessible and easier to use for participants in the general public, particularly those with low literacy or education.

The current study makes important contributions to the research on dementia in several ways. First, the shortened versions of the DKAS identified herein allow researchers and practitioners to incorporate additional constructs into their survey instruments. For example, in our future research we plan to collect data on the attitudes of bank employees towards customers who may have dementia in addition to the information gathered with the DKAS-s. Second, we expect the DKAS-s to be more efficient in field settings while retaining an acceptable level of test information among our survey’s target population: the general public.

The current study also has limitations that highlight possible directions for future research. First, we used purposive sampling to recruit participants, so our sample was not representative of the general population of Taiwan, although we tried to recruit participants from various backgrounds (i.e. college students, bank employees, and pharmacists) to represent the diversity of the population at large. However, as mentioned earlier, the ‘representativeness’ of the sample does not impact the estimates of the IRT parameters and thus should not significantly affect the performance of the assessment tool. Second, we did not have data to evaluate the convergent or divergent validity of the DKAS-s. Nevertheless, Annear et al. have demonstrated good concurrent validity between the original DKAS and the ADKS (Pearson correlation *r* = 0.56, *P* < .001) [12]. The DKAS2 has also been shown to have discriminative ability based on the finding of significant differences in knowledge of dementia among different occupational cohorts [13]. We believe that our DKAS-s also has good convergent validity because the scores of the shortened scale correlated strongly with those of the 25-item DKAS-TC scale (*r* > .95). Future studies could consider collecting responses on factors affecting dementia knowledge (e.g. self-reported attitudes towards dementia and data from educational interventions) in order to establish the criterion-related validity of the shortened scale. Third, although the range of difficulty of our DKAS-s items covers the majority of the latent trait levels of dementia knowledge, as does the range of difficulty of the items on the 25-item DKAS-TC (See Fig. 2), these items were less efficient in differentiating participants at either extremes of the amount of knowledge of dementia they had. Future studies on a shortened version of the DKAS-TC should consider adding items with a higher level of difficulty if the scale is used with medical or nursing professionals.

## Conclusions

In conclusion, using an IRT analytical approach, the current study developed a shortened version (DKAS-s) of the previously developed Chinese version of a 25-item dementia knowledge assessment tool (DKAS-TC). More specifically, we identified 16 items with above-average discriminative ability that retained at least 64% of the original total test information. It is our expectation that the DKAS-s will increase the utility of the assessment of dementia knowledge in both research and practice.

## Supplementary Information

**Additional file 1: Appendix 1.** Characteristics of participants.

**Additional file 2: Appendix 2: Table 1.** The short-form of the Dementia Knowledge Assessment Scale-Traditional Chinese version (DKAS-s). **Table 2.** Chinese/English statements about dementia in the short-form of Dementia Knowledge Assessment Scale-Traditional Chinese version (DKAS-s)

## Data Availability

The datasets analysed during the current study are available from the corresponding author on reasonable request.

## Abbreviations

ADKS: The Alzheimer’s Disease Knowledge Scale
DKAS: The Dementia Knowledge Assessment Scale (27 items)
DKAS2: The Dementia Knowledge Assessment Scale (25 items)
DKAS-TC: Traditional Chinese-language translation of the DKAS2
DKAS-s: The shortened scale of DKAS-TC (16 items)
CTT: Classical Test Theory
IRT: Item Response Theory
