# Supplementary material for: Developing a shortened version of the dementia knowledge assessment scale (DKAS-TC) with a sample in Taiwan: an item response theory approach
Source: BMC Geriatr. 2022 Nov 22;22:886. doi: 10.1186/s12877-022-03596-1 (PMC9682634; doi:10.1186/s12877-022-03596-1)
Supplement: Supplementary file 1 — Additional file 1: Appendix 1. Characteristics of participants. [file 12877_2022_3596_MOESM1_ESM.docx]

Appendix 1 Characteristics of participants

| Variables | N (%) | Banker | Pharmacist | Student | P value |
| --- | --- | --- | --- | --- | --- |
| Occupation |  |  |  |  |  |
| Banker | 102 (25%) | 102 | - | - |  |
| Pharmacist | 133 (32%) | - | 133 | - |  |
| Ungraduated Student | 175 (43%) | - | - | 175 |  |
| Age |  |  |  |  | <.0001 |
| <30 | 185 (47%) | 14 (14%) | 10 (7%) | 161 (100%) |  |
| 30-49 | 76 (19%) | 35 (35%) | 41 (31%) | - |  |
| 50-59 | 78 (20%) | 40 (39%) | 38 (29%) | - |  |
| ≥ 60 | 56 (14%) | 12 (12%) | 44 (33%) | - |  |
| Gender |  |  |  |  | .3099 |
| Male | 179 (46%) | 50 (50%) | 53 (40%) | 76 (47%) |  |
| Female | 213 (54%) | 50 (50%) | 78 (60%) | 85 (53%) |  |
